# The hidden link between HIV and cardiomyopathy: unraveling HIV's impact on the heart

**DOI:** 10.3389/fcvm.2025.1601430

**Published:** 2025-09-12

**Authors:** Toluwalase Awoyemi, Oluwaremilekun Zeth Tolu-Akinnawo, Andrew Greek, Oluwakemi Adenuga, Chukwuebuka Asogwa, Isaac Ekundayo, Olamide Odusola, Oyinlola Fasehun, Mercy Ajayi, Onyinye Ugoala, Luther-King Fasehun, Oluwaseun Dorcas Adeleke, Michael Angarone

**Affiliations:** ^1^ Northwestern University Feinberg School of Medicine, Chicago, IL, United States; ^2^ Meharry Medical College, Nashville, TN, United States; ^3^ Kansas City University College of Medicine, Joplin, MO, United States; ^4^ Olabisi Onabanjo University Teaching Hospital, Sagamu, Nigeria; ^5^ University of Ibadan College of Medicine, Ibadan, Oyo, Nigeria; ^6^ Faculty of Clinical Sciences, University of Ilorin, Ilorin, Nigeria; ^7^ University of Texas Rio Grande Valley-Knapp Medical Center, Weslaco, TX, United States; ^8^ University of Port-Harcourt Teaching Hospital, Port-Harcourt, Nigeria; ^9^ Department of Internal Medicine, Texas Tech University Health Sciences Center, Amarillo, TX, United States; ^10^ Department of Epidemiology, Columbia University Mailman School of Public Health, New York, NY, United States; ^11^ Sumy State University, Sumy, Ukraine; ^12^ Department of Infectious Disease, Northwestern University Feinberg School of Medicine, Chicago, IL, United States

**Keywords:** human immunodeficiency virus (HIV), cardiomyopathy, pathogens, myocardial damage, immune response, vasculitis, public health

## Abstract

This comprehensive review examines the complex relationship between human immunodeficiency virus (HIV) and cardiomyopathy, focusing on the underlying molecular mechanisms, clinical manifestations, diagnostic approaches, and treatment strategies. It highlights the significant global health burden posed by HIV and its potential to cause long-term cardiovascular complications. The review investigates the pathogenesis of HIV-associated cardiomyopathy. It elucidates the intricate cellular and molecular pathways involved, including the actions of neutrophils, monocytes, macrophages, and lymphocytes in cardiac inflammation. Key signaling pathways such as TNF-NF-κB and the caspase-1 inflammasome are detailed, as they contribute to cardiac infection and injury. The clinical manifestations of HIV-associated cardiomyopathy are discussed, including fatigue, dyspnea, peripheral edema, and arrhythmias. The review outlines essential diagnostic methods, highlighting the importance of cardiac biomarkers, electrocardiography, and imaging techniques such as echocardiography and cardiac MRI. Treatment strategies are explored, encompassing lifestyle modifications, pharmacological interventions, and advanced therapies. The review underscores the importance of addressing micronutrient deficiencies, particularly selenium, in the management of HIV-associated cardiomyopathy. It also discusses the role of antiretroviral therapy and the potential benefits of intravenous immunoglobulin therapy. Furthermore, this review addresses the evolving perspective on heart transplantation for individuals with HIV. It notes that while HIV was once considered a contraindication for transplantation, recent advancements in antiretroviral therapy have led to a re-evaluation of this stance. Finally, the review identifies future research directions, emphasizing the need for biomarkers to detect at-risk patients, exploration of nutritional factors predisposing individuals to cardiomyopathy, and further investigation into advanced therapies for HIV-associated cardiomyopathy. This review significantly enhances the understanding of HIV-associated cardiomyopathy, providing valuable insights for clinicians and researchers in the fields of infectious diseases and cardiology.

## 1 Introduction

Human Immunodeficiency Virus (HIV) is a major global health issue, affecting millions of individuals across diverse populations (1). Approximately 40 million people are living with HIV (2). The burden of HIV varies across regions and nations, with notable differences between gender, sexual orientation, age, and socioeconomic level (3). Specific high-risk populations that are disproportionally affected by HIV are men who have sex with men, African Americans, Latinos, and people who inject drugs (4). The high prevalence of HIV poses significant challenges for public health systems due to its potential to cause not only acute symptoms but also long-term health complications. People with HIV can remain asymptomatic for prolonged periods, contributing to their ongoing transmission and often delaying diagnosis and treatment (1). Clinical implications arise with immune suppression commonly seen in HIV and can include cardiovascular disease in chronic cases (5).

A growing area of interest is the link between HIV and the development of cardiomyopathy. Several cardiovascular pathologies have been associated with HIV, including dilated cardiomyopathy, myocarditis, pericardial effusions, and coronary artery disease (5). This comprehensive review seeks to explore the molecular and clinical pathways through which HIV influences cardiac health, with a focus on the mechanisms of myocardial injury and the long-term implications for patients. By elucidating these relationships, the review aims to contribute to a better understanding of how timely HIV diagnosis and treatment may mitigate cardiovascular risks.

## 2 Pathogenesis of HIV-associated cardiomyopathy

Cardiomyopathy can be induced or exacerbated by HIV through complex cellular and molecular pathways. At the molecular level, several cell types play key roles in cardiac inflammation. Neutrophils and monocytes are the first to arrive at the site of cardiac injury, releasing reactive oxygen species (ROS) and proteases aimed at removing the cause of the damage (6). This response can be self-destructive, potentially causing additional harm to the heart (7). Macrophages, once exposed to inflammatory signals, take on a proinflammatory role, which prolongs inflammation and heart tissue damage by releasing cytokines (7). These cytokines prompt fibroblasts and cardiomyocytes to become proinflammatory as well (6). Lymphocytes also help sustain the inflammation (6). After injury, cardiomyocytes release cytokines, including IL-6, which contributes to both cell growth (hypertrophy) and cell death (apoptosis), thereby exacerbating inflammation (6, 7). Cardiac fibroblasts, when exposed to inflammatory signals like TNF-α, adopt an inflammatory state and release cytokines such as IL-1 and IL-6, further fueling the ongoing inflammation (6).

Several signaling pathways are involved in these inflammatory processes, such as the TNF-NF-*κ*B pathway, which plays a role in heart infection and injury, and the caspase-1 inflammasome pathway, which is activated by oxidative stress (6, 7). Macrophages are central to these pathways, expressing receptors known as Toll-Like Receptors (TLRs) (6). TLRs detect molecules from pathogens or stressed cells, triggering a cascade of signals that lead to the production of proinflammatory cytokines through the NF-κB and mitogen-activated protein kinase (MAPK) pathways (6). This activation results in the generation of ROS and the release of cytokines, perpetuating the inflammatory response in the heart (6, 7). Moreover, the interplay between pathogens and host factors, including genetic susceptibility and comorbidities such as hypertension and diabetes, further complicates the pathogenesis of HIV-associated cardiomyopathy (HIVAC). This genetic variation (such as CCR% & CCR2 variants, CCL3L1, EFCAB14, CD101, UBE2V1, CXCL12) represents the major factor that contributes to the susceptibility and prognosis (7).

**Figure 1:**
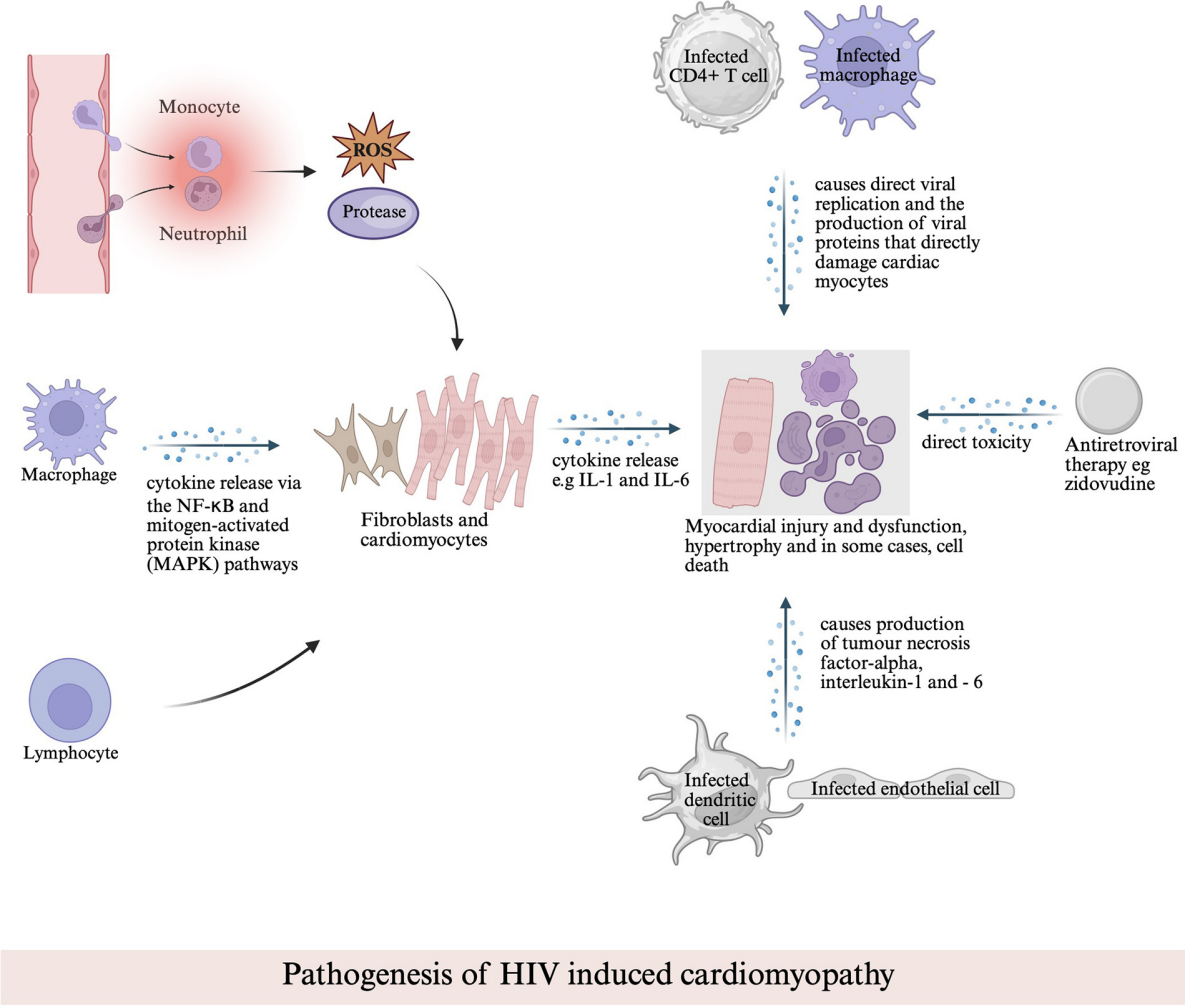
Pathogenesis of HIV-associated cardiomyopathy. This schematic illustrates the multifactorial mechanisms contributing to myocardial injury, including direct viral replication within cardiac cells, cytokine-mediated inflammation via NF-κB and MAPK pathways, immune cell–mediated damage, and antiretroviral drug–related toxicity.

HIVAC is multifactorial, involving viral-mediated mechanisms, immune dysregulation, and the indirect effects of opportunistic infections. HIV infects CD4+ T cells and macrophages in the heart, leading to viral replication and the production of viral proteins that directly damage cardiac myocytes (8). By infecting myocardial dendritic cells and endothelial, HIV induces abnormal inflammatory response which subsequently mediates chronic inflammation through production of tumor necrosis factor-alpha, interleukin-1 and—6, as well as other pro-inflammatory cytokines leading to myocardial injury and dysfunction (9). This chronic inflammation associated with HIV is thought to be responsible for increased myocardial fibrosis, and cardiac steatosis (10). Unfortunately, antiretroviral therapies (ARTs) itself can be potentially directly toxic to the myocardium with a common example being Zidovudine (AZT) which causes myocyte mitochondria damage which is reversible upon discontinuation of the medication (11). AZT has also been associated with an 8 times increased risk of HIVAC (12–15) (Figure 1).

Autoimmune mechanisms have also been implicated in the pathogenesis of HIVAC through HIV-induced B-cell stimulation leading to autoantibody production directed against the myocardium (16). This subsequently causes myocardial injury (myocarditis) and systolic dysfunction as antibodies damage the myocardium and contribute to inflammation and cardiac remodeling. Studies have shown evidence for cardiac-specific autoimmunity in HIV patients with symptomatic cardiac dysfunction (17). The prevalence of cardiac autoantibodies have been found to be significantly elevated in HIV-infected patients with left ventricular systolic dysfunction when compared to HIV-infected individuals without heart disease and HIV-uninfected controls (18). There have also been some evidence showing In review molecular mimicry between cardiac myocytes and HIV-1 core proteins p17 and p24 causing the immune system to mistakenly attack myocardial tissue (19).

## 3 Clinical manifestations and diagnosis

### 3.1 Signs and symptoms

Like other forms of cardiomyopathy, peripheral edema, involving the legs, ankles, feet, or abdomen, frequently results from fluid retention secondary to compromised myocardial function. Fatigue, disproportionate to exertion levels, is a common clinical manifestation of HIVAC, often accompanied by dyspnea, particularly during exertion or in recumbency (20). Arrhythmias may present as palpitations or a fluttering sensation, reflecting electrical disturbances in the heart (20, 21). Although chest pain is not universally observed, it can emerge during physical exertion or periods of heightened stress. Additionally, symptoms such as dizziness, light-headedness, and syncope may arise due to impaired cerebral perfusion (20, 21). A persistent cough, especially with frothy or hemoptysis-like sputum, may signify heart failure progression (20). Moreover, reduced exercise tolerance and lymphadenopathy, notably in the cervical region, can also be correlated with HIVAC.

### 3.2 Arrhythmias and sudden cardiac death in HIVAC

Cardiac electrical disturbances are a recognized complication of HIVAC. Among people living with HIV (PLWH), electrocardiographic abnormalities, including QTc prolongation, fragmented QRS complexes, and nonspecific ST–T wave changes, are common. QTc prolongation, reported in approximately 18%–29% of PLWH, is associated with heightened risk for malignant ventricular arrhythmias such as torsades de pointes (22). Contributing mechanisms include chronic systemic inflammation, autonomic dysfunction, myocardial fibrosis, electrolyte imbalances, and ART–related blockade of cardiac potassium channels. Although rare, Brugada-pattern electrocardiographic changes have been described in HIV, potentially reflecting ion channel modulation by viral proteins or ART-related effects, which may predispose to polymorphic ventricular tachycardia (23).

These arrhythmic substrates contribute to a markedly increased burden of sudden cardiac death (SCD) in this population (24). SCD ranks as the third leading cause of mortality in PLWH and occurs at rates up to 4.5-fold higher than in HIV-negative individuals (25). Risk is highest among those with poorly controlled HIV, particularly with high viral loads, low CD4 counts, or concomitant cardiovascular comorbidities but is still present in mild HIV disease (25). Myocardial fibrosis, frequently observed in cardiac magnetic resonance imaging studies of PLWH, is thought to serve as an anatomical substrate for re-entrant ventricular arrhythmias leading to SCD (26).

Given these findings, systematic rhythm surveillance in HIVAC, including baseline and periodic ECG assessment, may facilitate early detection of arrhythmia-prone patients and inform risk stratification for implantable cardioverter-defibrillator (ICD) consideration.

### 3.3 Diagnostic methods

Once a diagnosis of HIVAC is confirmed, identifying the underlying etiology becomes essential, as it informs subsequent therapeutic strategies. Blood tests frequently reveal elevated levels of cardiac biomarkers, including B-type natriuretic peptide (BNP) and troponin, which serve as indicators of cardiac stress (27).

Additionally, electrocardiographic abnormalities, such as widened QRS complexes, prolonged QT intervals, and various arrhythmias, further substantiate the presence of cardiac pathology. Transthoracic echocardiography (TTE) is an invaluable tool for the early detection of cardiac dysfunction in asymptomatic HIV-positive individuals and acquired immunodeficiency syndrome (AIDS) patients, especially in patients with advanced disease as evidenced by low CD4+ counts (28, 29).

To ensure a comprehensive evaluation, all patients should undergo coronary angiography to exclude ischemic cardiomyopathy and assess potential interventions. This procedure is standard for new-onset heart failure but is particularly significant in the HIV population due to their elevated risk of coronary artery disease (CAD), which persists independently of traditional cardiovascular risk factors (29). While TTE remains the gold standard for diagnosing heart failure, advanced imaging techniques such as cardiac magnetic resonance imaging (CMR) are increasingly utilized. However, CMR is not typically employed in the initial diagnostic work-up; its current role is primarily focused on investigating disease prevalence and pathogenesis, particularly in asymptomatic HIV-positive individuals. Importantly, CMR has revealed increased rates of myocardial fibrosis and lipid deposition within this population, although its utility for individual diagnosis remains limited (29).

### 3.4 Emerging biomarkers

Growing evidence supports the utility of biomarkers such as Galectin-3 (Gal-3), soluble ST2 (sST2), and soluble urokinase plasminogen activator receptor (suPAR) for risk stratification in heart failure, including HIV-associated cardiomyopathy. Elevated Gal-3 levels have been observed in PLWH compared to uninfected controls, suggesting ongoing myocardial fibrosis and inflammation (30). Similarly, observational studies report increased plasma concentrations of sST2, Gal-3, and growth differentiation factor-15 (GDF-15) in PLWH, although direct prognostic data linking these markers to HIVAC outcomes remain limited (31). These biomarkers hold promise for early detection and prognostication in this population. Future studies are warranted to validate these biomarkers in HIVAC populations and explore their integration into existing risk stratification models.

### 3.5 Cardiac electrophysiological risks in HIV-associated cardiomyopathy

Importantly, PLWH are also at increased risk for electrophysiological abnormalities, including QT interval prolongation and Brugada-pattern electrocardiographic changes, which contribute to a heightened risk of sudden cardiac death (25).

QT prolongation in HIV may be multifactorial, driven by chronic inflammation, ART effects, electrolyte imbalances, and direct myocardial involvement. Brugada-pattern ECG changes, though less common, have been increasingly reported and may reflect underlying ion channel dysfunction induced by HIV or its treatment.

Recognition of these electrophysiological alterations is crucial, as they may precede malignant ventricular arrhythmias and SCD. Integration of biomarker profiling with detailed cardiac electrophysiological assessment could enhance risk stratification and guide timely interventions to reduce mortality among PLWH.

## 4 Discussion

### 4.1 Treatment and management

#### 4.1.1 HIV prevention

Reducing the prevalence of HIVAC starts with the prevention of HIV. Safe sex practices, such as correct condom use, are highly effective in preventing HIV. The in-review utilization of pre-exposure prophylaxis (PrEP) and post-exposure prophylaxis (PEP) have been recommended by the Centers for Disease Control and Prevention (CDC) to greatly reduce the risk of contracting HIV. Abstaining from injecting drugs or otherwise using clean needles will minimize the risk of HIV through drug use (32).

#### 4.1.2 Lifestyle modifications

The intricate interplay between micronutrient deficiencies and cardiovascular health in individuals living with HIV, highlights the need for targeted nutritional interventions. In HIV patients, micronutrient deficiencies are commonly observed in this population due to factors such as malabsorption, chronic diarrhea, and wasting syndrome (33). These deficiencies exacerbate oxidative stress, leading to the formation of free radicals and subsequent myocardial damage, which is linked to the development of HIVAC (34). Notably, selenium deficiency has been identified as a significant contributor to cardiomyopathy in untreated HIV-positive individuals (35). A prospective study involving 416 HIV-positive individuals in Rwanda found that reduced serum selenium levels were associated with nearly double the likelihood of developing cardiomyopathy, according to multivariate analysis (36). Supporting these findings, animal models have demonstrated that selenium-deficient mice exhibit increased susceptibility to myocardial damage (18). Given this evidence, dietary intake of selenium-rich foods—such as Brazil nuts, seafood, meat, poultry, organ meats, cereals, and dairy products—should be encouraged among individuals with HIV particularly those at risk for HIVAC (37).

In addition to addressing micronutrient deficiencies, adopting broader preventive strategies is crucial. The recently updated Life's Essential 8 (LE8) metrics, outlined by the American Heart Association (AHA), have demonstrated significant benefits in reducing cardiovascular disease risk and promoting overall cardiovascular health (38). The components of LE8 include physical activity, body mass index, blood pressure, diet, nicotine exposure, blood lipids, blood glucose, and sleep. A study evaluating the association between LE8 scores and cardiovascular and all-cause mortality revealed that higher LE8 scores were strongly and inversely correlated with cardiovascular disease mortality (38). Therefore, promoting measures that improve LE8 scores is particularly important in high-risk populations, such as those with HIV, to enhance cardiovascular health.

#### 4.1.3 Atrial fibrillation in PLWH

Meta-analyses indicate an elevated risk of atrial arrhythmias in people living with HIV (incidence ∼6.4 per 1,000 person-years; relative risk 1.35 vs. controls) (39). Risk factors include lower CD4 counts and higher viral loads (39). HIV-positive patients without severe immunosuppression demonstrate similar success rates after AF ablation compared to matched controls, with pulmonary vein reconnection being the predominant cause of recurrence; atrial voltage substrate appears comparable (40, 41). Management should align with standard AF guidelines (rate/rhythm control and anticoagulation), with special attention to drug–drug interactions between ART and anticoagulants or antiarrhythmics. Recent data suggest a lower bleeding risk with apixaban compared to warfarin or rivaroxaban in this population (42). Given the elevated arrhythmia risk in HIVAC, periodic ECG monitoring and rhythm surveillance should be considered part of comprehensive management.

#### 4.1.4 Pharmacological interventions

Early initiation of beta-blocker and ACE inhibitor therapy offers promise in preventing the progression of subclinical HIVAC into severe systolic dysfunction by addressing common pathological mechanisms such as afterload reduction and sympathoadrenal modulation (43). In parallel, for HIVAC, ART has been shown to play a multifaceted protective role in the myocardium, preventing direct HIV-associated damage. Notably, case reports have documented the regression of cardiomyopathy in both adults and children undergoing ART (44, 45).

Different ART classes have divergent cardiovascular profiles. Protease inhibitor–based regimens, particularly those containing lopinavir/ritonavir and abacavir, have been associated with increased cardiovascular risk (e.g., MI relative risk ∼1.41 vs. non-PI regimens) (46). Conversely, earlier initiation of ART (post-2015) appears associated with reduced incidence of hypertension, hyperlipidemia, and coronary artery disease (47). Although ART may improve endothelial function, residual risk persists, highlighting the need for cardiotoxicity-aware ART selection (48) (Table 1).

**Table 1:** Summary of cardiovascular effects and clinical considerations for antiretroviral therapy (ART) drug classes. This table outlines common ART classes, representative drugs, associated cardiovascular effects, and relevant clinical considerations to guide therapy selection in people living with HIV at risk for cardiovascular disease.

ART class	Example drugs	Cardiovascular effects	Clinical considerations
NRTIs	Tenofovir, Abacavir, Zidovudine (AZT)	Abacavir linked with ↑ myocardial infarction (MI) risk; Zidovudine causes mitochondrial toxicity → cardiomyopathy	Avoid abacavir in high cardiovascular (CV) risk; monitor cardiac function if using AZT
NNRTIs	Efavirenz, Nevirapine	Efavirenz: possible QT prolongation; Nevirapine: ↑ lipid levels	Monitor ECG if high arrhythmia risk; check lipid profile
PIs	Lopinavir/ritonavir, Atazanavir, Darunavir	↑ MI risk (especially with lopinavir/ritonavir); dyslipidemia; insulin resistance	Avoid in uncontrolled CV risk; implement lipid-lowering strategies
INSTIs	Dolutegravir, Raltegravir, Bictegravir	Generally neutral CV profile; some weight gain	Preferred in patients with high CV risk
Entry Inhibitors	Maraviroc, Enfuvirtide	Minimal direct CV impact; Maraviroc may improve endothelial function	Safe in most CV patients; monitor for rare hypotension

In addition to ART, intravenous immunoglobulin (IVIG) therapy has demonstrated significant improvements in cardiac function in pediatric HIV patients. A retrospective study of 49 children revealed reductions in left ventricular wall thickness and peak wall stress, along with enhanced left ventricular contractility and fractional shortening following IVIG administration (49). Monthly IVIG infusions have also been effective in addressing subclinical cardiac abnormalities in HIV-infected children (50, 51). However, controlled trials in adult populations are lacking, indicating a gap in research and the need for further investigation to optimize treatment strategies for HIVAC (52, 53).

Further supporting these efforts, clinical trials such as the ENCHANTMENT HIV study are exploring innovative treatments like sacubitril/valsartan to prevent HIV-related cardiac remodeling (35). These trials highlight the ongoing need to develop tailored therapies for cardiovascular complications in PLWH.

Addressing micronutrient deficiencies, particularly selenium, is also crucial in managing HIVAC. Selenium, essential for the production of glutathione peroxidase, protects the heart from oxidative stress caused by free radicals. Supplementing selenium in deficient patients, either preventively or to correct existing deficiencies, has shown clinical benefits (54). In a case series of three HIV-positive patients with selenium deficiency, daily supplementation with 200 µg resulted in echocardiographic and clinical improvements as selenium levels normalized over three months (55). This emphasizes the importance of addressing nutritional deficiencies in managing HIVAC.

**Figure 2:**
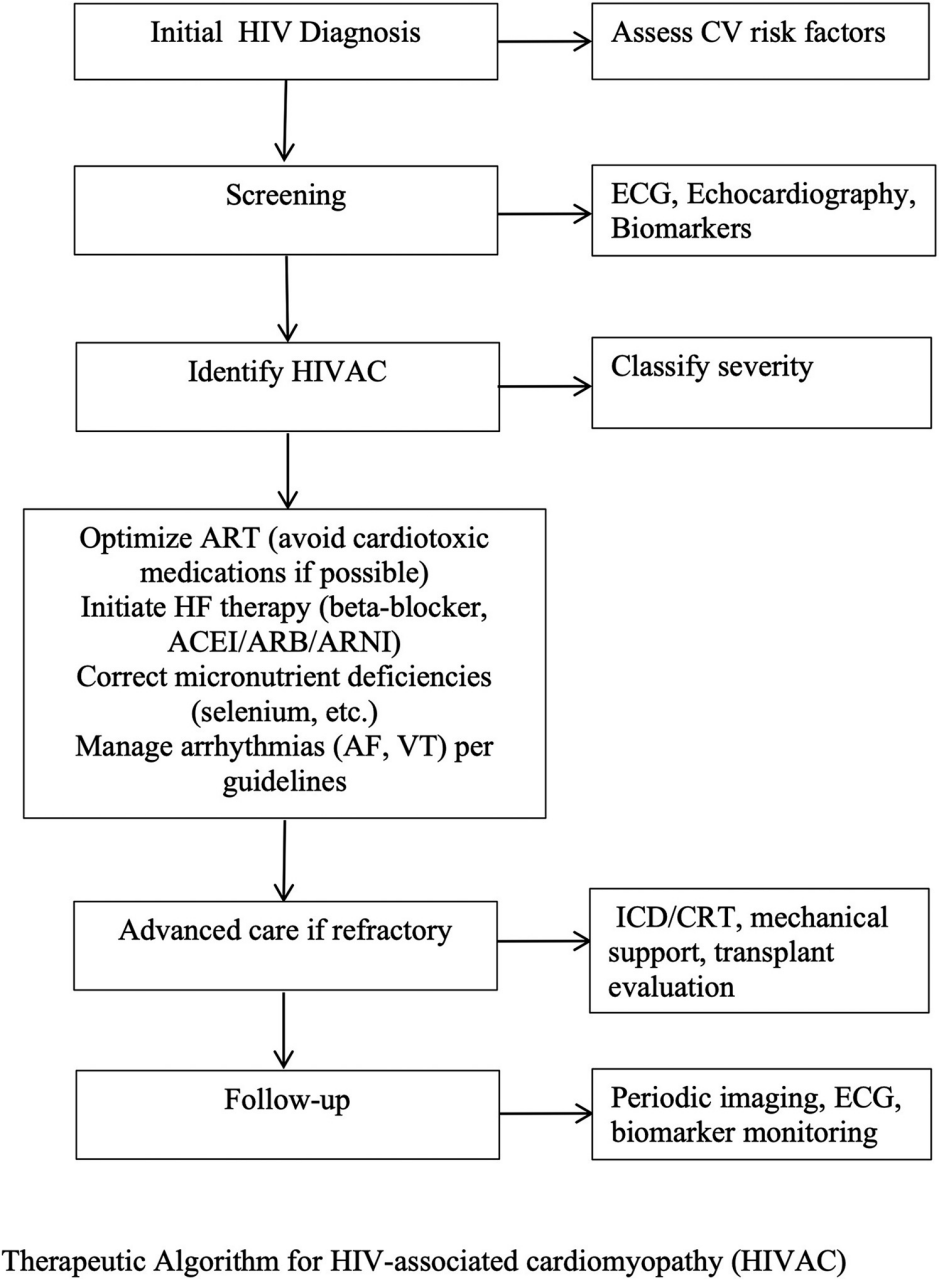
Therapeutic algorithm for HIV-associated cardiomyopathy (HIVAC). This stepwise approach begins with initial HIV diagnosis and cardiovascular risk assessment, followed by targeted screening and identification of HIVAC with severity classification. Management includes optimization of antiretroviral therapy, initiation of guideline-directed heart failure therapy, correction of micronutrient deficiencies, and arrhythmia management. Advanced interventions such as ICD/CRT, mechanical circulatory support, or heart transplantation are considered for refractory cases, with ongoing follow-up incorporating imaging, ECG, and biomarker monitoring.

Moreover, in patients with cardiomyopathy as a result of hepatitis C virus (HCV), cardiac complications may arise independently of HIV. Antiviral treatments, such as sofosbuvir and daclatasvir, provide an effective strategy to mitigate the risk of cardiomyopathy in individuals with HCV (56). This underscores the importance of a comprehensive, multidisciplinary approach in managing cardiomyopathy in patients with co-infections, incorporating both ART and cardiovascular interventions (Figure 2).

#### 4.1.5 Heart transplantation

Mechanical support devices and cardiac transplantation represent definitive treatment options for HIVAC (57). However, their application within HIV-infected populations has been historically constrained. HIV infection was once regarded as a contraindication to mechanical support and transplantation due to concerns about increased mortality rates associated with end-stage heart failure (58). Nevertheless, advancements in ART have prompted a re-evaluation of this stance. The United Network for Organ Sharing (UNOS) now recommends that asymptomatic HIV-infected individuals should not be automatically excluded from heart transplant consideration solely based on their HIV status (59).

Traditionally, the high mortality rates linked to HIV, coupled with apprehensions regarding immunosuppression, have reinforced the perception of HIV infection as a contraindication for heart allotransplantation (44). However, there remains a notable paucity of data concerning the long-term outcomes for PLWH who have undergone heart transplantation. While earlier studies reported unfavorable outcomes for PLWH receiving cardiac transplants, more recent literature indicates that there has been no significant increase in organ rejection or deterioration of immunosuppressive sequelae in this patient population (45).

Furthermore, existing consensus data suggest that PLWH may experience cardiovascular improvement with ART, leading to the consideration that select patients could be suitable candidates for heart transplantation (59). Evidence from case series and small cohort studies conducted in the USA and Canada supports this notion, demonstrating favorable outcomes for HIV-infected patients, with survival rates comparable to those of their HIV-uninfected counterparts for up to five years post-transplant (59–61).

Despite these promising findings, a recent survey of cardiac transplantation centers revealed that 57% of programs continue to view HIV infection as a contraindication to transplantation (62). This hesitance stems from concerns about the limited availability of donor organs, the potential for post-transplant immunosuppression to exacerbate the In review progression to advanced HIV and AIDS, and the risk of drug interactions between HIV treatments and immunosuppressive therapies post-surgery. However, there are limited data showing similar short-term and moderate-term survival after heart transplantation for HIV positive recipients when compared to HIV-negative recipients (59). Similar data has been seen in liver and kidney transplants as well (63).

**Figure 3:**
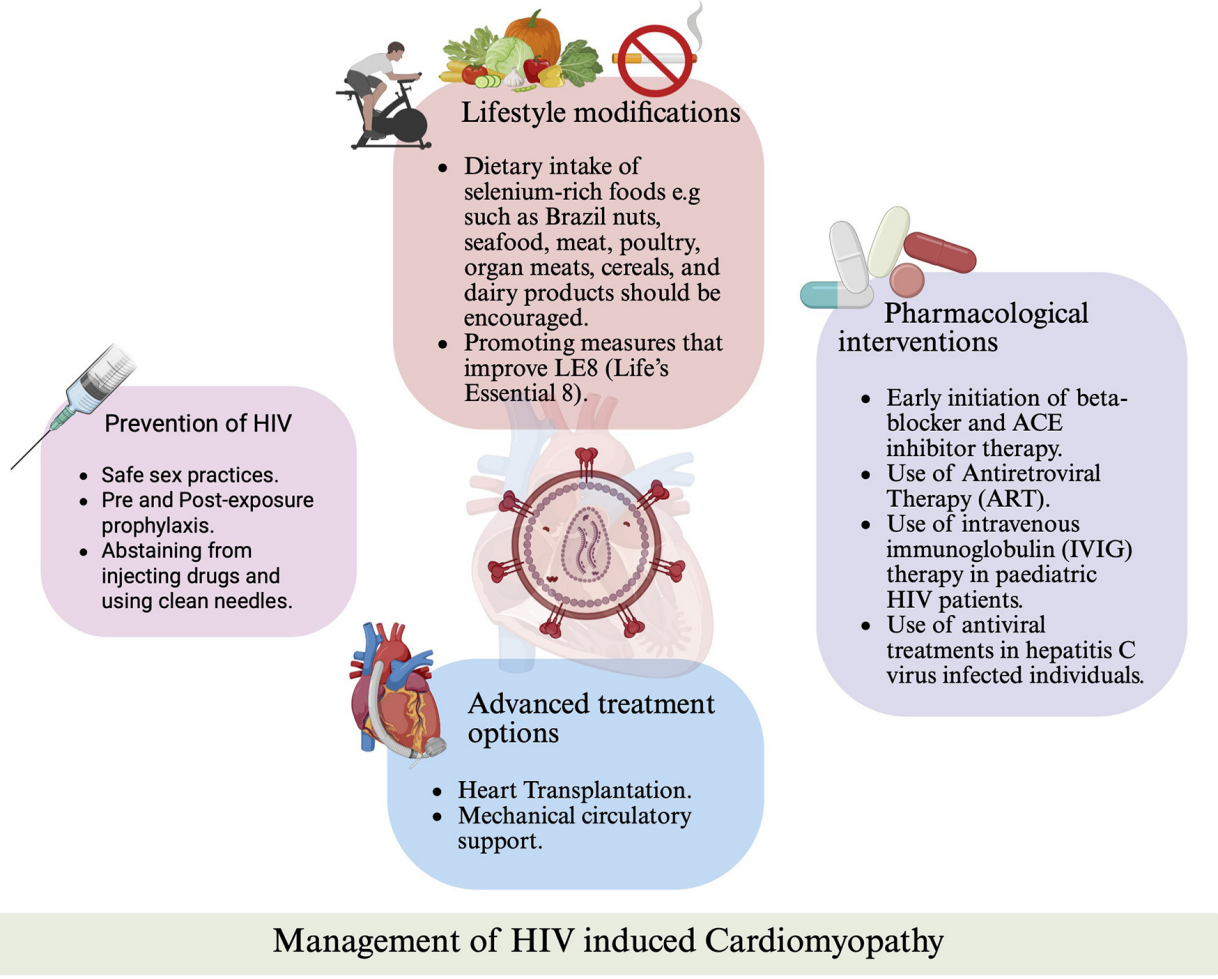
Management strategies for HIV-associated cardiomyopathy. Overview of preventive measures, lifestyle interventions, pharmacologic treatments (including ART, beta-blockers, ACE inhibitors, and IVIG), and advanced therapies such as mechanical circulatory support and heart transplantation.

While additional data are necessary to fully establish the safety and efficacy of these advanced therapies, recent findings underscore the importance of considering individuals with HIVAC for advanced treatment options, including transplantation and mechanical circulatory support (Figure 3).

### 4.2 Future directions and research opportunities

Further research is imperative to identify and address several critical limitations in the field of HIVAC. A limited understanding of the relevant pathogenesis provides challenges in vaccine development. Limitations also include the need for biomarkers to detect patients at risk of developing HIVAC, and the exploration of nutritional and micronutrient deficiencies that may predispose individuals to cardiomyopathy following HIV acquisition, as evidenced by the role of selenium (51, 64). Additionally, cardiac remodeling drugs warrant further investigation, as emerging evidence suggests that certain medications may facilitate the reversal of myocardial defects, as demonstrated in the ENCHANTMENT HIV clinical trial (35).

### 4.3 Implications for public health policy

It is imperative for policy analysts and decision-makers to gather more data by promoting research focused on HIVAC. Such efforts will facilitate prevention strategies, early diagnosis, risk stratification, and the development of treatment guidelines. The lack of clarity regarding management protocols remains a significant challenge, making it essential to prioritize this issue given the global burden of HIVAC (18).

## 5 Conclusion

In conclusion, HIV-associated cardiomyopathy poses a significant public health challenge that requires increased awareness and targeted interventions. Individuals with HIV face a heightened risk for cardiomyopathy, worsened by factors like micronutrient deficiencies and inflammatory responses, as well as the effects of highly active antiretroviral therapy. Despite advancements in treatments such as cardiac transplantation and mechanical support, barriers like historical biases and immunosuppression concerns persist. Further research is vital to clarify mechanisms, identify biomarkers for early detection, and explore tailored treatment options. By integrating cardiovascular health into HIV management and fostering interdisciplinary collaborations, we can improve patient outcomes and mitigate the cardiovascular complications associated with HIV.
